# Polyethylenimine based magnetic nanoparticles mediated non-viral CRISPR/Cas9 system for genome editing

**DOI:** 10.1038/s41598-020-61465-6

**Published:** 2020-03-12

**Authors:** S. S. Rohiwal, N. Dvorakova, J. Klima, M. Vaskovicova, F. Senigl, M. Slouf, E. Pavlova, P. Stepanek, D. Babuka, H. Benes, Z. Ellederova, K. Stieger

**Affiliations:** 1 The PIGMOD center, Institute of Animal Physiology and Genetics, v. v. i., The Czech Academy of Sciences, Libechov, Czech Republic; 2 Institute of Molecular Genetics, The Czech Academy of Sciences, Praha 4, Czech Republic; 3 Institute of Macromolecular Chemistry CAS, Heyrovského nám. 2, 162 06 Prague 6, Czech Republic; 4 Department of Ophthalmology, Justus-Liebig-University, 35392 Giessen, Germany

**Keywords:** Biotechnology, Cell delivery

## Abstract

Clustered regularly interspaced short palindromic repeats-associated protein (CRISPR/Cas9) system has become a revolutionary tool for gene editing. Since viral delivery systems have significant side effects, and naked DNA delivery is not an option, the nontoxic, non-viral delivery of CRISPR/Cas9 components would significantly improve future therapeutic delivery. In this study, we aim at characterizing nanoparticles to deliver plasmid DNA encoding for the CRISPR-Cas system in eukaryotic cells *in vitro*. CRISPR/Cas9 complexed polyethylenimine (PEI) magnetic nanoparticles (MNPs) were generated. We used a stable HEK293 cell line expressing the traffic light reporter (TLR-3) system to evaluate efficient homology- directed repair (HDR) and non-homologous end joining (NHEJ) events following transfection with NPs. MNPs have been synthesized by co-precipitation with the average particle size around 20 nm in diameter. The dynamic light scattering and zeta potential measurements showed that NPs exhibited narrow size distribution and sufficient colloidal stability. Genome editing events were as efficient as compared to standard lipofectamine transfection. Our approach tested non-viral delivery of CRISPR/Cas9 and DNA template to perform HDR and NHEJ in the same assay. We demonstrated that PEI-MNPs is a promising delivery system for plasmids encoding CRISPR/Cas9 and template DNA and thus can improve safety and utility of gene editing.

## Introduction

There is a new trend in the development of gene therapies for monogenic diseases investigating the application of the CRISPR/Cas9 system for editing the genome in a precise, sequence-dependent manner, resulting in a permanent change of the genomic information at the target site. The CRISPR/Cas9 (clustered regularly interspaced short palindromic repeats-associated proteins) technology has become one of the most significant genomic engineering tools. Since 2012, this system was widely used in different cell lines and organisms due to its high efficiency, simplicity and versatility for creating insertions and deletions via error-prone non-homologous end joining (NHEJ) or high-fidelity homology-directed repair (HDR) using a template DNA^1–4^. Furthermore, it represents the most promising strategy in current gene therapy applications and is used to generate animal models to enable xenotransplantation and other biomedical applications^5^.

Generally, components of the CRISPR/Cas9 system can be introduced into cells as DNA in the form of a plasmid or as mRNA, or as a ribonucleotide protein (RNP) complex. Due to it’s stability, ease to handle and low cost effect, the plasmid DNA format is the most attractive and approachable mode for delivery. However, in recent year there is rapid advancement in novel biomaterials for efficient and gene vehicles to cross cellular barriers under physiological conditions and promote transgene expression can be attained^6^.

The transfer of the genetic information into target cells is a major challenge in gene therapy, and the effectiveness of gene expression is dependent on multiple factors involved in the processes of cellular uptake, intracellular release and nuclear entry^7^. One of the most popular modes of delivery for genetic material including DNA sequences encoding CRIPSR/Cas9 is the use of viral vectors such as adeno-associated virus (AAV) or lentivirus (LV) vectors^8^. Despite having several advantages like high transduction efficiency and the potential for long term transgene expression, viral vectors have certain disadvantages like increased immunogenicity (LV), prevailing cytotoxicity (LV), a small packing size (AAV), expensive large scale production (all vectors), and the risk of integrating viral sequences into the target genome (LV)^8^.

In order to overcome such difficulties, non-viral delivery systems for CRISPR/Cas9 are a promising alternative. Importantly, plasmid DNA ensures a temporary burst of transgene expression, thus limiting the activity of the CRISPR/Cas9 system and reducing off target toxicity when comparing it to long-term viral vector mediated transgene expression^9^. Several non-viral delivery vehicles have been developed for delivery of CRISPR/Cas9 such as human serum albumin nanoparticles (NPs), lipid NPs and gold NPs^10–12^. Furthermore, recently, viral vector capsids have been used to couple RNPs to it for NP like gene transfer into cells^13^. Despite the potential advantages for genome editing applications compared to viral vectors, the efficiency of NP based gene transfer is still not optimal. NPs have great advantages due to their nanoscale size, high surface to volume ratio, good colloidal stability and biocompatibility. Among them, magnetic nanoparticles (MNPs) have unique properties such as high magnetization values and the ability to pass cellular barriers. Additionally, they have been used for magnetic resonance imaging (MRI), gene delivery, in magnetic hyperthermia therapy and tissue repair. Therefore, the functionalized MNPs are expected to be useful as a new gene delivery tool^14–16^. Polyethylenimine (PEI), a cationic polymer is known as a widely used transfection reagent in molecular biology^16,17^, and a dispersant in nanotechnology^18^. PEI forms a positively charged complex with DNA, which binds to anionic cell surface residues and enters the cell via endocytosis^16,17^, keeping the dispersed state in the solution^18^. Several surface modified NPs, such as cationic arginine gold NPs, cell-penetrating peptides, CRISPR-PAsp (DET) gold NPs, graphene oxide–polyethylene glycol–PEI have been previously tested for delivering CRISPR/Cas9 components into different cell types^19–24^. Recently even PEI–MNPs were shown to improve efficiency of commonly used transfection reagent (DreamFect Gold) to deliver CRISPR/Cas9 for gene editing in porcine fibroblast for generating genetically modified pig models^24^. But this transfection combination does not have the potential to be applicable in therapeutic *in vivo* genome editing.

The long term goal of our study is to develop MNPs that allow efficient therapeutic genome editing applications. This requires a robust chemical characterization, prove of stability and colloidal dispersion, as well as efficient internalization into human cells, first in *in vitro* study, followed by *in vivo* studies. Here, we generated and characterized MNPs with regard to their physical and chemical properties and their potential to deliver CRISPR/ Cas9 plasmids into cells without the help of Lipofectamine like transfection reagents.

CRISPR/Cas9-PEI-MNPs complexes were designed to be internalized by cells via endocytosis due to the cationic PEI, which after endocytosis triggers endosomal disruption and causes the release of CRISPR/Cas9-PEI-MNPs into the cytoplasm. A HEK293 cell line expressing the modified traffic light reporter (HEK293-TLR-3) system to quantitatively determine the DNA repair by NHEJ or HDR through flow cytometry and confocal microscopy analysis was used^4^. Several biophysical characterizations and cytotoxicity assays were carried out in order to prove the efficacy and safety of MNPs.

## Materials and methods

Chemicals: Ferrous chloride (FeCl_2_), ferrous sulphate (FeSO_4_.7H_2_O), sodium hydroxide (NaOH), PEI (MW 25,000) NaOH were procured from Sigma Aldrich.

### Cell Culture

A stable HEK293-TLR3 cell line previously developed by us was used^4^. It expresses the traffic light reporter (TLR-3) system containing the blue florescent protein (BFP) gene to quantify non-homologous end joining (NHEJ) and green florescent protein (GFP) gene to quantify homology directed repair (HDR) events. The cell line was maintained in DMEM supplemented with 10% FBS, 200 mM Ala-glutamine (Sigma Aldrich), and 50 U/mL Gentamicin (Life Technologies), and cultured at 37 °C with 5% CO2 incubation. Two days prior to magnetofection, the cells were seeded into 12- well plates (TPP, Sigma Aldrich) at a density of 5 × 10^5^ cells/well in 1 mL of supplemented media. The degree of cell confluency at the time of transfection was ∼50–60%. Cells in the absence of MNPs were also cultured and in this plate identified as untreated cells or negative control. The cells transfected with 2 µg of CRISPR-Cas plasmid by Lipofectamine 2000 (Invitrogen, Thermofisher Scientific), were treated as positive control.

### CRISPR-Cas9-gRNA-mRFP Construct and Template Construct for HDR

Cloning and design of all CRISPR plasmid constructs as well as template was described previously^4^. Briefly, the Cas9 expression vector, pSpCas9 (BB)-2A-Puro (PX459) V2.0, was purchased from Addgene (plasmid 62988). The guide RNA T3: 5′-GGTGAGCTCTTATTTGCGTAGGG-3′ for targeting the traffic light reporter (TLR3) system was cloned into the px459 expression vector using In-Fusion Cloning Kit (Clontech), according to the manufacturer’s instructions^4^. Plasmid without guide RNA marked T0 was also generated for negative control purposes. The puromycin resistance gene was exchanged with mRFP gene using In-Fusion Cloning Kit. The plasmid containing mRFP gene was purchased from Addgene (plasmid 13032). The donor template with corrected GFP gene sequence (~1000 bp, start codon absent) was amplified using Phusion polymerase (NEB), and the PCR product was further cloned into the TOPO TA Cloning Kit (Life Technologies) for the generation of the plasmid donor template^4^. Plasmids were prepared by using GenElute HP Plasmid Maxiprep Kit. The concentration of plasmid DNA (pDNA) solution was determined by using the nanodrop method.

### Synthesis of magnetic nanoparticles

Fe_3_O_4_ nanoparticles were synthesized by a chemical co-precipitation method^25^. In brief, FeCl_3_.6H_2_O and FeSO_4_.7H_2_O were dissolved in 100 ml deionized water with a molar ratio of 2:1 under vigorous mixing at 80 °C. Then, NaOH was slowly added into the mixture until the pH reached up to 11. On addition of NaOH, the precipitate turned black, indicating the formation of Fe_3_O_4_ nanoparticles. The mixture was stirred vigorously for 15 min and aged for 30 min at 70 °C and then the reaction mixture was cooled down to room temperature. Fe_3_O_4_ nanoparticles were separated by magnetic separation and washed by deionized water repeatedly to remove the impurities (Supplementary Fig. S3). Finally, these synthesized Fe_3_O_4_ MNPs were dispersed in deionized water.

### Synthesis of PEI coated MNPs and complexed with CRISPR-Cas 9 plasmid

PEI-coated MNPs were formed at N/P ratio 10 (nitrogen in PEI-coated MNPs/phosphorus in DNA) by mixing volumes of the aqueous solution of PEI, DNA and MNPs. Briefly, 50 μl of PEI (40 μg/mL) was mixed with 2 µg of DNA and incubated at room temperature for 30 min. The resulting CRISPR/Cas9-PEI plasmid complexes were then incubated with 100 μl of bare MNP (200 μg/mL) in water for 1 h. The PEI-MNPs has been considered as bare NPs (without plasmid) was also incubated with at the same time. After 1 h of incubation with MNPs and PEI + CRISPR-Cas 9 plasmid, the final mixture was termed as PEI-MNPs, T0-CRISPR/Cas9-PEI-MNPs (MNPs containing the CRISPR-Cas9-gRNA-mRFP and template plasmids with scrambled gRNA T0) and T3-CRISPR/Cas9-PEI-MNPs (MNPs containing the CRISPR-Cas9-gRNA-mRFP and template plasmids with target specific gRNA T3), respectively. The final MNPs to CRISPR-Cas 9 plasmid weight ratio was always kept at 10 in all the synthesis experiment.

### Physicochemical characterization

The size distribution and colloidal stability measurements were performed with a Zetasizer Nano-ZS instrument, Model ZEN3600 (Malvern Instruments, UK). The dispersions in Millipore water were sonicated for 1–2 minutes. The particle size was measured at 25 °C at a scattering angle 173°. The zeta potential measurements were performed using a universal zeta dip cell.

The morphology and the particle size of the MNPs were analyzed by transmission electron microscopy (TEM) using Tecnai G2 Spirit Twin 12 microscope. 3 μl of MNPs in aqueous solutions were dropped onto a copper TEM grid (300 mesh). The filter paper was used to remove the excess of solution. The imaging of dried MNPs was done in bright field at 120 kV.

Infrared spectra were measured by a spectrometer Spectrum 100 T FT-IR (PerkinElmer) equipped with deuterated triglycine sulphate detector using the attenuated total reflectance (ATR) technique. Four scans per spectrum (650–4000 cm^−1^) at the resolution of 4 cm^−1^ were measured.

### Magnetofection

PEI-MNPs, T0-CRISPR/Cas9-PEI-MNPs, and T3-CRISPR/Cas9-PEI-MNPs (2 μg DNA/well) were incubated with cultured cells in serum free media for 30 min followed by addition of 10% FBS. Under some plates (wells) a magnet was placed for 1 h to test if magnetofection will help the complex enter the cells. The cells were then further incubated for 24 h in the absence of magnetic field, washed, and cultured with a fresh media for another 48 h at 37 °C in a 5% CO_2_ humidified atmosphere. At the end of cultivation, the cells were washed 2 times with PBS, trypsinized and re-suspended in 800 μl media to have the final volume of 1 ml.

### FACS analysis

After 3 days post transfection cells were washed with PBS 2–3 times to remove the bounded MNPs. The cells were trypsinized (0.05%) then collected and re-suspended in fresh media. FACS analysis of mRFP/BFP/GFP-positive events were performed using a LSR II cytometer (Becton-Dickinson). The gene expression efficiency, which measured the percentage of RFP-positive cells, was determined by normalizing the number of cells expressing RFP against untreated cells. The presented data were obtained from three independent experiments performed in duplicate.

### Confocal microscopy

TLR-HEK cells were seeded at a density of 4 × 10^5^ cells/well on 8-well (Nunc Lab-Tek Chambered Coverglass (Thermo Scientific)) the day before use. Cells were incubated with PEI-Coated-MNPs-CRISPR/Cas9 plasmid complexes (1 µg DNA/well) in serum free media for 30 min followed by addition of 10% FBS via magnetic assisted transfection (magnetofection), in which a magnet was placed underneath the cells for 1 h to facilitate the localization of MNPs to the adhered cells during *in vitro* gene delivery. The cells were incubated for 24 h with these magnetic complexes. After 24 h media was replaced by fresh medium and further incubated for 3 days. The cells were further observed on confocal microscope Leica TCS SP5.

### Cell proliferation assay

For evaluation of cytotoxicity the AlamarBlue cell viability assay (Thermo Fisher Scientific) was performed. TLR-HEK-cells were seeded on 12 well TPP plate until the cell confluency reached up to 70–80%. The cells were transfected with Lipofectamine or T3-CRISPR/Cas9-PEI-MNPs. Then 100 μl of alamarBlue reagent (10×) was added to the cells, and incubated for 2 h at 37 °C before measuring the absorbance at 535/600 nm.

### Agarose gel electrophoresis

2 µg of CRISPR-Cas9 plasmid DNA was complexed with PEI, and a total volume of 50 µL was mixed with 10 µL loading buffer from Invitrogen (Carlsbad, CA, USA) and applied to a 1% TAE agarose gel containing 7 µL gel red dye (0.5 mg/ml). For the CRISPR/Cas9 plasmid labeled T0, 1 µl of NdeI restriction enzyme (Invitrogen) was used. For the CRISPR/Cas9 plasmid labeled with T3 + template, 1 µl of restriction enzyme NdeI and 1 µl of restriction enzyme NotI for (Invitrogen) template cleavage were added. This was followed by incubation for 12 hours at 37 ° C. Electrophoresis was carried out with a current of 80 V for 2 h in TAE as running buffer.

## Results and discussion

### Synthesis of PEI-Coated/CRISPR plasmid nanoparticles complexes

**Figure 1:**
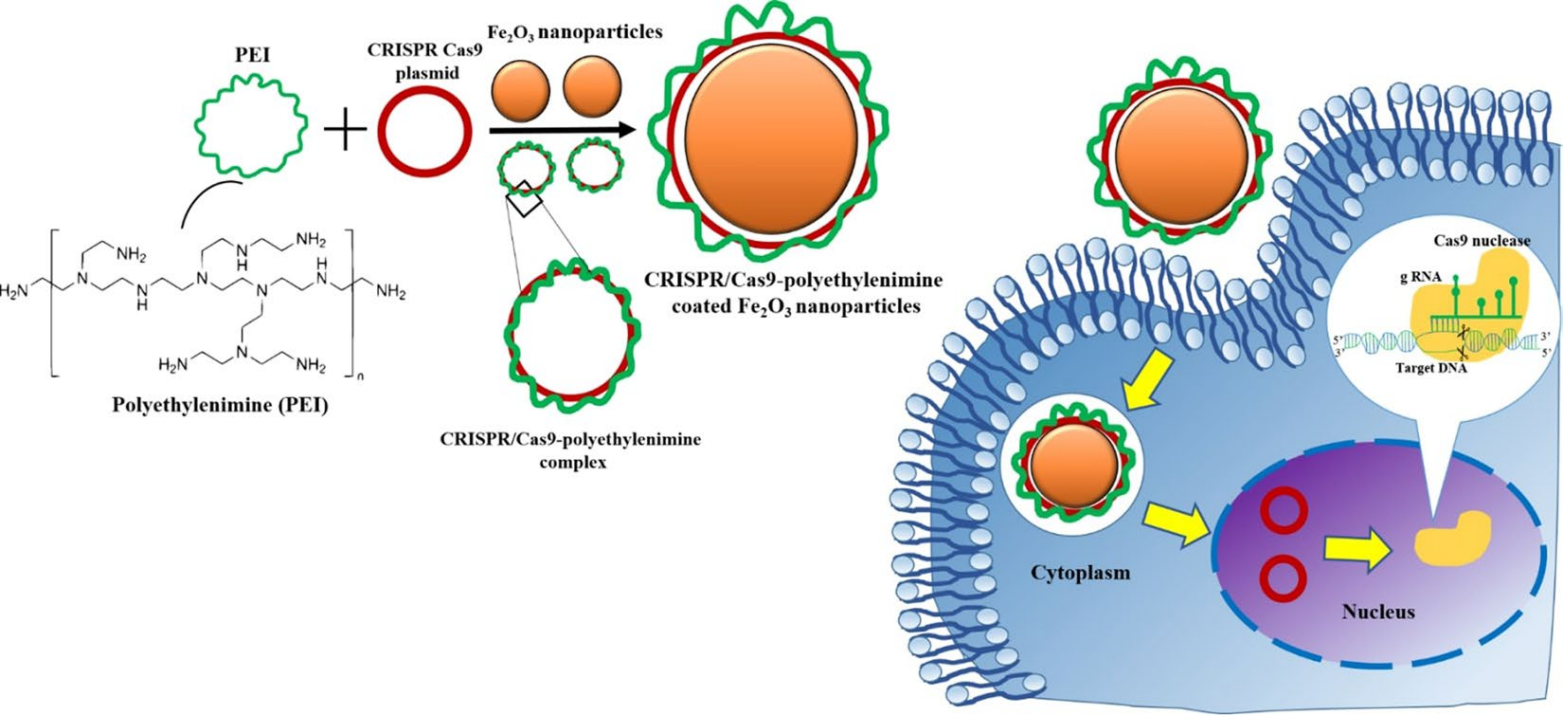
Schematic representation of the synthesis of magnetic nanoparticles (MNPs). MNPs are generated by co-precipitation and further complexed with CRISPR/Cas9 plasmids to form a complex, which is transferred into HEK 293-TLE-3 cell line by magnetofection. The nanoparticles are internalized by the cells via endocytosis due to the cationic PEI, which after endocytosis triggers endosomal disruption and causes into the cytoplasm.

The DNA/PEI complexes are capable of escaping from endosomes or preventing endo-lysosome formation by the proton sponge effect^26^. In order to improve the transfection efficiency, surface modification of MNPs was done by conjugating PEI positively charged NH_2_ groups to the negatively charged phosphate group of the DNA **(**Fig. 1**)**. Herein, the MNPs have sufficient electric potentials at the surface, capable of being combined with transfection complex CRISPR/Cas9-PEI via electrostatic adsorption to meet the requirements of magnetofection. Thus, the preparation of MNPs can be independent of the plasmid used. This CRISPR/Cas9-PEI-MNPs complex was then transfected into a stable TLR-HEK293 cell line previously generated by us to test the efficacy of genome editing^4^.

### Hydrodynamic particle size distribution and colloidal stability

**Figure 2:**
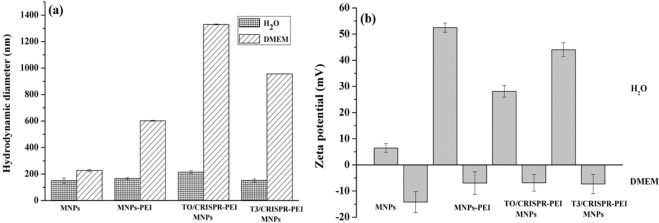
Hydrodynamic diameter and zeta potential of magnetic nanoparticles (MNPs). (**a**) Hydrodynamic diameter of MNPs in the presence of water and DMEM at pH 7.0 (mean ± S.D.; n = 3), (**b**) Zeta potential of T3 and T0-CRISPR/Cas9-PEI-MNPs in the presence of water and DMEM at pH 7.0 (mean ± S.D.; n = 3).

The physicochemical characterization of MNPs and PEI-MNPs indicating the size, shape and charge was carried out to determine the potential of this system to enter the cell and nucleus. Dynamic light scattering (DLS) of MNPs was analysed to determine the hydrodynamic particle size for all of our formulations as shown in Fig. 2a. For the uncoated MNPs, we observed particle size to be around 150 nm in diameter. After surface functionalization of MNPs by PEI, the particle size was slightly increased to 166 nm. This increase in size could be explained by the nature of PEI, which has more hydrophilic groups to interact with water molecules after suspension. The surface functionalization by PEI increased the stability, improved colloidal dispersion and reduced aggregation of MNPs. After addition of the CRISPR/Cas plasmid, the particle size was further increased to 214 nm in case of T0 plasmid not containing the gRNA sequence, and decreased to 152 nm in case of the T3 plasmid containing the gRNA sequence. This change in particle size is likely due to the presence of both PEI and DNA, which is involved in association and dissociation of water molecules creating a hydration shell around MNPs. These results are in accordance with Kami *et al*. and Arsianti *et al*., although our results shows smaller hydrodynamic diameters illustrating better colloidal stability of PEI coated MNPs^27,28^.

Additionally, when these magnetic complexes were suspended in 10% FBS DMEM medium, notably larger sizes (226, 602, 1330 and 956 nm) of NPs were observed due to aggregation (respectively for empty MNP, empty PEI-MNP, and both the T0-CRISPR/Cas9-PEI-MNPs and T3-CRISPR/Cas9-PEI-MNPs complexes). This probably attributes to the presence of serum containing salt, even though it is believed that serum adsorption onto PEI-coated magnetic complexes improves the vector dispersion in DMEM through steric stabilization. Also, the binding between MNPs and biomacromolecules could be realized for example by ion exchange binding, i.e. positively charged groups (e.g. amino groups) of the biomacromolecules (e.g. proteins) bind to negatively charged groups (e.g. carboxyl groups) of the MNPs. A common feature of the MNPs showing aggregation in FBS is the presence of reactive groups in the coating. These results are in agreement with previously published data by Chen *et al*. and Chithrani *et al*.^29,30^ on MNPs suspended in DMEM. The polydispersity indices obtained for all samples were around 0.3, thus showing that the NPs are highly monodisperse. In any case, these MNPs have been sonicated well before transfection to cells to avoid further aggregation due to sedimentation.

### Surface charge distribution and stability of magnetoplexes by zeta potential

To evaluate the stability of the MNPs, zeta potential analysis was performed. The zeta potential of different MNP configurations at pH 7 is presented in Fig. 2b. The zeta potential of empty MNP, empty PEI-MNP and both the T0-CRISPR/Cas9-PEI-MNPs and T3-CRISPR/Cas9-PEI-MNPs complexes possessed a positive charge in water (16, 52, 28 and 44 mV respectively) due to the presence of PEI on the outer layer of the MNPs. Moreover, the values of zeta potential of all four magnetoplexes with positive charge became negative when the MNPs were suspended in 10% DMEM containing serum (−13.5, −6.43, −6.34 and −7.36 mV for empty MNP, empty PEI-MNP and both the T0-CRISPR/Cas9-PEI-MNPs and T3-CRISPR/Cas9-PEI-MNPs complexes, respectively), indicating adsorption of serum components onto to the surface of the MNPs. As PEI is positively charged and DNA is negatively charged, a positive and negative zeta potential can be used to indicate whether PEI or DNA dominates at the MNP complex surface. The T0-CRISPR/Cas9-PEI-MNPs and T3-CRISPR/Cas9-PEI-MNPs magnetoplexes were assembled by coupling bare MNPs with PEI/plasmid complexes that have a positively charged zeta potential in water (around 16 mV), indicating the attachment of the positively charged PEI/plasmid complexes at 28 and 44 mV for the T0 and the T3 CRISPR/Cas9 plasmid, respectively. These results are in accordance with previously reported findings, yet our presented work shows better colloidal stability as compared with other studies^27–30^.

### Transmission electron microscopy

**Figure 3:**
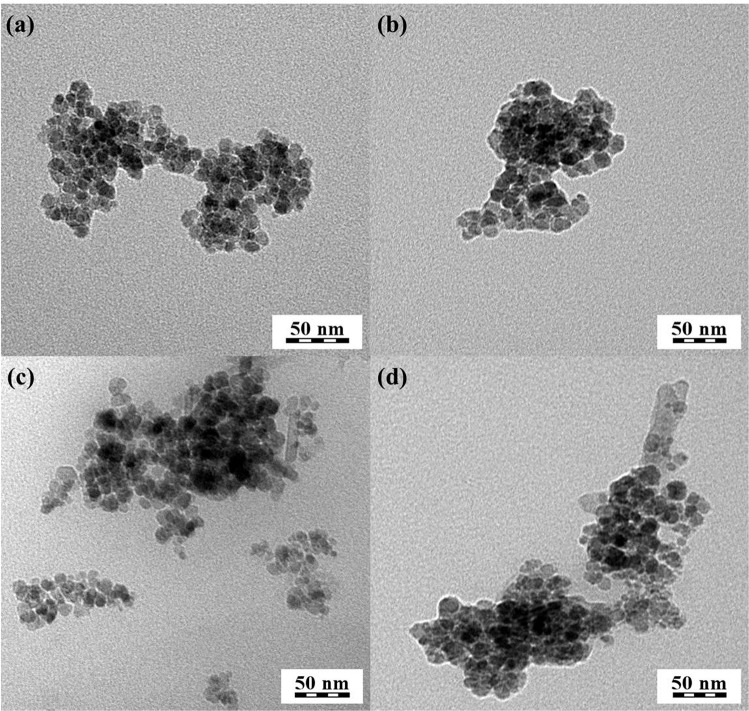
Ultrastructure of magnetic nanoparticles (MNPs). TEM micrographs of magnetic-nucleic acid complexes: (**a**) empty MNPs, (**b**) empty PEI-MNP, (**c**) T0-CRISPR/Cas9-PEI-MNPs 0, and (**d**) T3-CRISPR/Cas9-PEI-MNPs, respectively.

TEM showed that the MNPs prepared by co-precipitation (Fig. 3) had an average diameter around 20 nm for all formulations. The aggregation of empty PEI-MNP (Fig. 3b) is slightly increased, which could be attributed to the PEI and may be due to the interparticle magnetic interation of MNPs. Interestingly, T0-CRISPR/Cas9-PEI-MNPs and T3-CRISPR/Cas9-PEI-MNPs (Fig. 3c,d) exhibited more pronounced aggregation, which was likely due to the electrostatic interaction of aggregated particle composed of several discrete nanoparticles. These results were in accordance with the DLS data, which confirmed the narrow particle size distribution of the aggregates that was observed by TEM. Similar results were obtained by Arsianti *et al*. and Namgung *et al*.^28,31,32^. Previous studies indicated that the optimal size of nanoparticles for effective gene delivery is to be smaller than 50–100 nm^28,31^. Therefore, the magnetoplexes derived from PEI coated MNPs acquired optimal size and morphology for efficient gene delivery.

### Fourier transform infra-red spectroscopy

**Figure 4:**
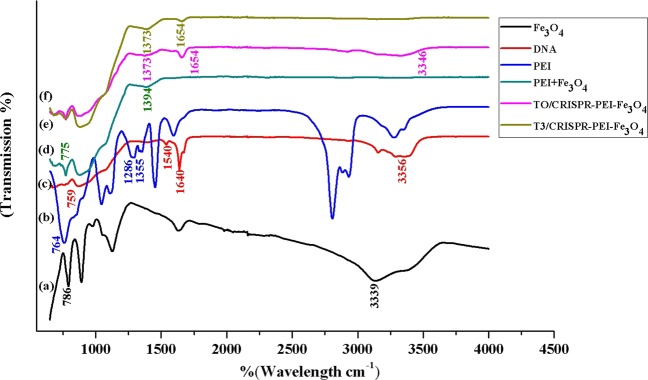
FTIR spectroscopy of the generated magnetic nanoparticles (MNPs). (**a**) MNPs, (**b**) plasmid DNA, (**c**) PEI, (**d**) PEI-MNPs, (**e**) T0-CRISPR/Cas9-PEI-MNPs and (**f**) T3-CRISPR/Cas9-PEI-MNPs.

FTIR analysis was performed (Fig. 4) in order to confirm the presence of functional groups on T3 and T0-CRISPR/Cas9-PEI-MNPs. The FTIR spectra of empty MNP, T0-CRISPR/Cas9-PEI-MNPs, and T3-CRISPR/Cas9-PEI-MNPs exhibit a broad absorption peak from 2972 to 3339 cm^−1^, which is due to the stretching vibration of the −OH group arising from the hydroxyl group of nanoparticles, DNA and water molecule^33,34^. The absorption vibration peak of Fe ^2+^ O^2−^ bonds at 604 cm^−1^ can be seen at 786 cm^−1^ in pure Fe_3_O_4_ which signifies the presence of a magnetite phase^26,33^. The characteristic peak of pure DNA appears at 1640 cm^−1^ corresponds to the amide I: C=O, C−N, and N−H. The band at 1540 cm^−1^ corresponds to CO-N bond, 759 cm^−1^ corresponds to out of plane bending vibration of =C−H and −CH_2_^35^. PEI showed absorption peaks at 1355 cm^−1^ and 764 cm^−1^ corresponding to C-N stretching and bending vibrations, respectively. The aromatic ether C-O-C peak appears in between the ranges of 1050–1150 cm^−1^ in PEI^36^. In PEI-Fe_3_O_4_, the characteristic peaks corresponding to PEI were clearly observed at 775 for cm^−1^ which corresponds to C-N bending. The 1286 cm^−1^ of the aromatic ether C-O-C was shifted to 1394 cm^−1^ due to surface coating as observed in the FTIR spectrum. We therefore confirmed that the PEI layer was coated on the surface of the MNPs.

The other characteristic peaks for DNA in the MNPs-PEI-pT0 and -pT3 was observed at 1654 cm^−1^, which corresponds to C=O, C−N, and N−H. The CO-N band at 1580 cm^−1^ has been shifted to either higher or lower frequencies due to the interaction of MNPs with PEI and DNA. Additionally, the characteristic peaks of PEI corresponding to C-N stretching and bending vibrations have been shifted and were observed at 1373 cm^−1^ and 792 cm^−1^ ensuring that PEI-DNA makes a composite-like structure with Fe_3_O_4_ molecules through chemical bonding.

### Binding affinity and magnetic response of magnetoplexes and their stability in culture medium

**Figure 5:**
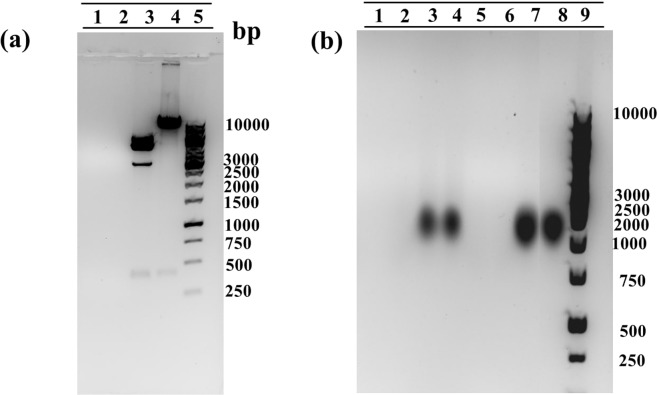
Gel electrophoresis retardation assay. (**a**) PEI-Coated/CRISPR plasmid nanoparticles complexed at an N/P ratio 10 Lane 1: T3-CRISPR/Cas9-PEI-MNPs, Lane 2: T0-CRISPR/Cas9-PEI-MNPs, Lane 3: T3 pure plasmid with restriction enzyme (R.E.), Lane 4: T0 pure plasmid with R.E, Lane 5: ladder 1 KB (**b**) Characterization of stability in FBS medium of magnetofectins Lane 1: T3-CRISPR/Cas9-PEI-MNPs with R.E., Lane 2: T0-CRISPR/Cas9-PEI-MNPs with R.E., Lane 3: T3 pure plasmid with R.E., Lane 4: T0 pure plasmid with R.E., Lane 5: T3-CRISPR/Cas9-PEI-MNPs without R.E., Lane 6: T0-CRISPR/Cas9-PEI-MNPs without R.E., Lane 7: T3 pure plasmid without R.E., Lane 8: T0 pure plasmid without R.E., Lane 9: ladder 1 KB.

A gel retardation assay was used to evaluate the binding affinity of the plasmid that was bound to MNPs. PEI (MW 25,000) has abundant amine groups and can completely bind oligonucleotides or plasmids to neutralize the negatively charged phosphate groups of plasmid DNA, resulting in the loss of electrophoretic mobility of the plasmid DNA. The results as shown in Fig. 5a revealed that plasmids complexed with PEI as the core were tightly bounded to Fe_3_O_4_ MNPs (lane 1 and lane 2), showed absent migration under the influence of the electric field in electrophoresis even after treating them overnight with specific restriction enzyme (R.E.) Nde I (for T0 and T3 plasmid) and Not I for (T3 plasmid with template). The results confirm that due to the binding of PEI with pDNA-CRISPR/Cas9 and MNPs, these magnetoplexes were sedimented along with the MNPs in the form of pDNA-CRISPR/Cas9-PEI-MNPs ternary complexes. Furthermore, lanes 3 and 4 are used as the controls of pure plasmids T0 and T3. These results are in accordance with previously published data^37,38^.

To accomplish higher gene transfection efficiency for *in vivo* applications, gene delivery carrier must shield pDNA from enzymatic degradation in serum. This could be achieved by using most prevailing method by physically shielding the pDNA through the electrostatic binding of the cationic polymer PEI. In order to confirm the protection of pDNA against serum enzymes such as nucleases, we incubated the complex of PEI-Coated/CRISPR plasmid nanoparticles in 10% fetal bovine serum (FBS) at 37 °C for 24 h. As shown in Fig. 5b (Lane 7 and Lane 8), naked pDNA was degraded within 24 h but pDNA complexed with PEI-coated magnetic particles survived beyond 24 h (Lane 5 and Lane 6). Therefore, the magnetofections of PEI-Coated/CRISPR plasmid NPs protect pDNA efficiently and successfully against serum enzymes at the N/P ratio of 10. Likewise, Namgung *et al*. synthesised BPEI-SPION complexed with pDNA and incubated it in 30% FBS for 24 h, which showed no degradation of pDNA even at the N/P ratio of 2^31^. Ma *et al*. has complexed pDNA-PEI-MNPs at different N/P ratios and analysied these complexes with and without serum on agarose gel electrophoresis to check the level of complexity of pDNA-PEI-MNPs, which revealed similar results^38^.

### Cell viability assay

**Figure 6:**
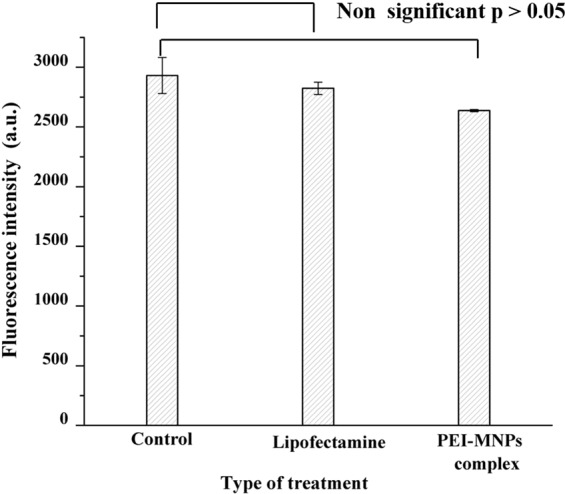
Cellular viability assay. Treated and control (untreated) cells at 24 h post treatment with Lipofectamine and PEI-MNPs complex without plasmid. No significant differences in cellular viability compared to untreated cells with p > 0.05 were detected.

The cellular metabolic activity and population of live/dead cells relative to the untreated blank TLR HEK293 cells 24 h post magnetofection with MNPs complexes without DNA is shown in Fig. 6. The percentage (%) of viable cells was calculated by fraction of F sample and F negative control relative to fraction of F positive control and F negative control multiplied by 100%. It was observed that there was no significant drop in the cell metabolic activity i.e. from 96% (for lipofectamine (p value is 0.54)) to as low as 90% (for MNPs (p value is 0.12)) showing only a marginal drop in cell activity indicating that these MNPs exhibits good biocompatibility toward TLR HEK299 cells. One possible explanation for the marginal drop in cytotoxicity is due to the presence of PEI which functions as a proton sponge after cell uptake and eventually destroys the endosome/lysosome^16^. Similar to our observations, by studying the survival rate of the cells after magnetofection, Hryhorowicz *et al*. showed no differences in the level of cell viability between magnetofection and Lipofection in porcine fibroblast cell^5^. Kami *et al*. has evaluated the cytotoxicity of PEI max and PEI max-nanoparticles by Alamar Blue assay. No significant differences in cell viability between mouse embryonic carcinoma cells (CL6) treated with PEI max and those with PEI max-nanoparticles after 48 h of PEI max or PEI max-nanoparticle exposure^27^. In contrast, Arsianti *et al*. used baby hamster kidney cells (BHK2I) to study the cell viability for 48 h post-magnetofection and observed a significant drop in the cell metabolic activity from 97% to 60% for cells that were transfected with either DNA/PEI + MNP, PEI/MNP + DNA/PEI, or PEI/MNP + DNA + PEI vectors^28^. While metabolic activity is not equal to cell viability, these data suggest an effect on the viability, which may be explained by different experimental setups among the different groups, leading to slightly contradictory results.

### DNA repair ability of magnetoplexes

**Figure 7:**
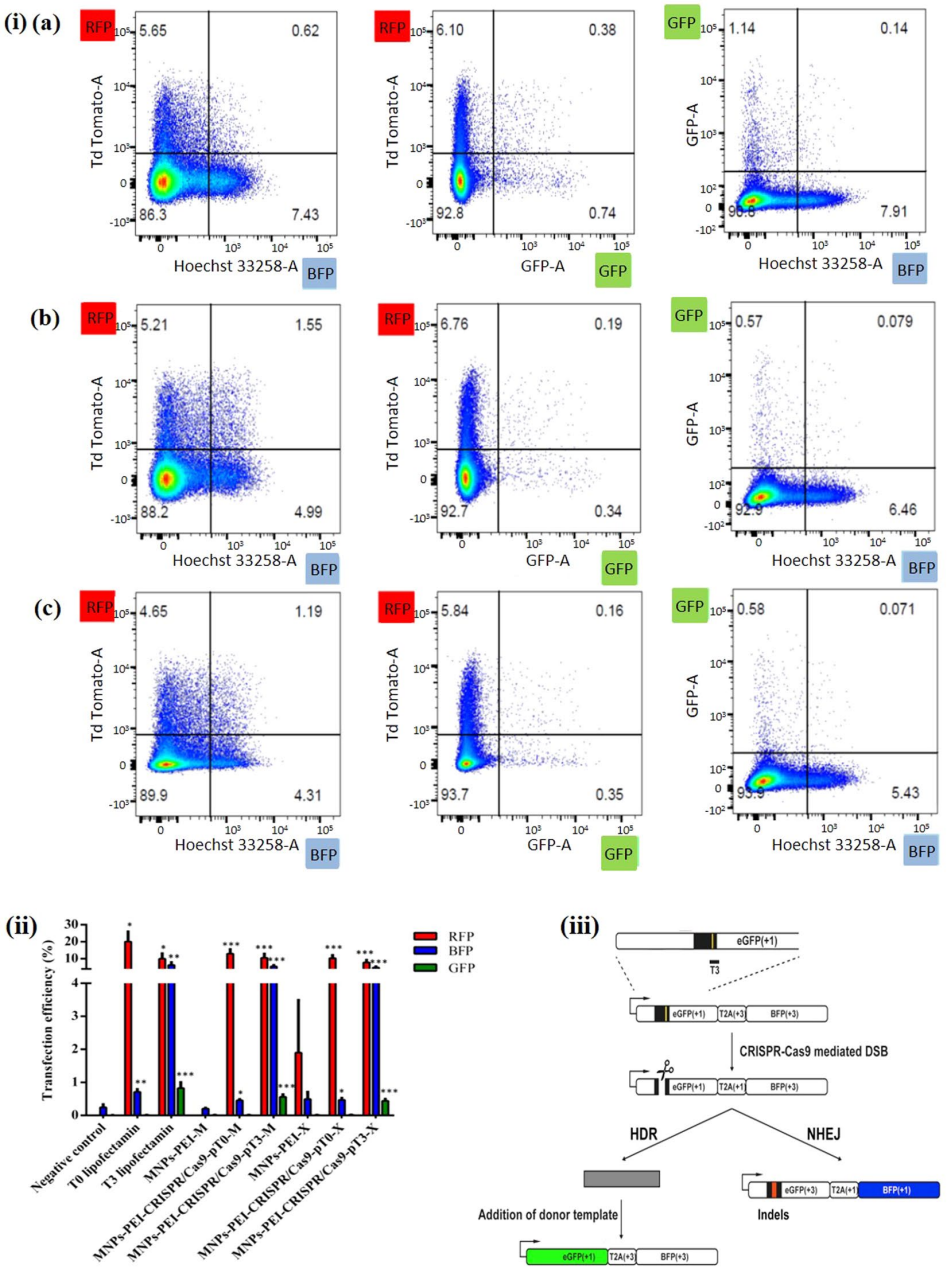
Quantification of genome editing results in the HEK293-TLR-3 cell line using magnetic nanoparticles (MNPs). (i) FACS representative data of the respective samples and controls with T3 positive control (Lipofectamine), T3-CRISPR/Cas9-PEI-MNPs-M magnetofected, T3-CRISPR/Cas9-PEI-MNPs-X non-magnetofected. (ii) Quantification of the transfection efficacy of NHEJ and HDR values compared with blank, and magnetofected (M) compared with non-magnetofected (X) transfections of the different complexes indicated. Statistical analysis: paired t test, *p < 0.01; **p < 0.001; ***p < 0.0005, ****p < 0.00005 (iii)Design of CRISPR-Cas9 guide RNA Targeting TLR3 Sequence: TargetT3 around the stop codon and I-SceI site (yellow line) have been designed for the specific cleavage within the GFP sequence in the TLR3 system. Depending on the addition of the donor templates, DSBs can be repaired through either NHEJ (BFP) or HDR (GFP). T, target^4^.

The DNA repair activities in cells based on fluorescence microscopy and fluorescence-activated cell sorting (FACS) was monitored using a modified TLR system in the TLR HEK293 cell line **(**Fig. 7iii). This TLR system contains an expression cassette of a non-functional GFP gene, including an ISce-I site and a stop codon, and a non-functional BFP gene shifted by 2 base pairs in a reading frame. Depending on the addition of the donor templates, site specific DSBs caused by CRISPR/Cas9 can be corrected through either NHEJ (BFP) or HDR (GFP)^4,27^.

**Figure 8:**
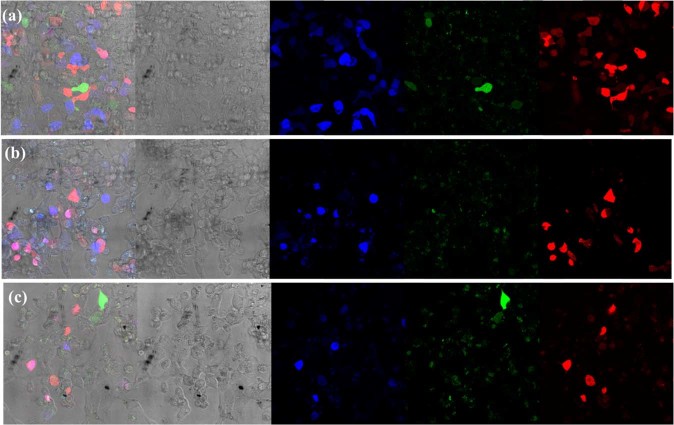
Fluorescent microscopy image of HEK293-TLR-3 cells transfected by T0 and T3 CRISPR/Cas9-PEI-MNPs complex at N/P ratio 10 for 72 h. The percentage of transfection efficiency is determined by mRFP positive cells (**a–c**), the type of DNA repair mechanism NHEJ and HDR due to the presence of TLR3 sequence is visualized by BFP and GFP respectively, (**a**) T3 positive control transfected with Lipofectamine, (**b**) T3-CRISPR/Cas9-PEI-MNPs magentofected and (**c**) T3-CRISPR/Cas9-PEI-MNPs non-magnetofected.

As our MNPs were endowed with promising attributes like CRISPR/Cas9 plasmid condensation ability and cell viability, we next focused our attention to test the potential of magnetofection towards its role as gene delivery vehicle in achieving enhanced transfection efficacy compared to classic lipofection with magnetofection with and without the influence of a magnetic field. To evaluate magnetofection, magnetoplexes were added to HEK293-TLR-3 cells and were incubated for 24 h at 37 ° C with and without the presence of a magnetic plate. Subsequently, the magnetoplexes containing media were replaced by fresh media containing 10% FBS to experience further levels of editing efficacy of NHEJ and HDR repair. The magnetoplexes were incubated for another 72 h followed by FACS and confocal microscopy (Figs. 7 and 8, and Supplementary Fig. S1).

We used PEI-MNPs magnetoplexes with two different CRISPR/Cas9 T0 and T3 for transfection in combination with an applied magnetic field that facilitates vector internalization, endosomal escape and protects the nucleic acid against nuclease degradation. To examine the transfection efficiency of CRISPR/Cas9 magnetoplexes, we magnetofected these MNPs into the HEK293-TLR3 stable cell line and counted fluorescing cells by FACS. By measuring the RFP-positive cells, we demonstrated transfection efficacy of 20% by using Lipofectamine as transfecting agent. In comparison, T0-CRISPR/Cas9-PEI-MNPs complexes showed the transfection efficacy around 13 and 10% respectively with and without the influence of magnetic field (Fig. 7(ii)). The levels of genome editing efficiency at the target site for NHEJ was explored by measuring the expression of blue fluorescence in BFP-positive cells for NHEJ. When lipofectamine was used as a positive control for T3-CRISPR/Cas9 plasmid 6% of HEK293-TLR3 cells underwent NHEJ. However, for T3-CRISPR/Cas9-PEI-MNPs magnetoplexes the percentage of NHEJ was observed to be around 5.4% for magnetofected and 4.7% for non-magnetofected cells. On the other hand, in order to observe HDR the presence of template is mandatory which makes this repair less prominent. In comparison with Lipofectamine (positive control), where 0.8% of the cells showed HDR, the T3-CRISPR/Cas9-PEI-MNPs magnetoplexes in presence of template showed GFP fluorescence expression at 0.5% and after magnetofection at 0.4% (Fig. 7(ii)).

### Effect of magnetic field on the magnetofection

Previously, several groups showed that magnetic field can help accumulate magnetoplexes in proximity of the cells during transfection^6,31^. In order to explore whether the applied magnetic force causes only the accumulation of MNPs near the cells or whether it can also influence the endocytosis and intracellular pathways, we carried out the transfection experiments with and without the influence of magnetic-field. As shown above, the NHEJ repair activity was observed to be around 5.4% and 4.7% for transfection by T3-CRISPR/Cas9-PEI-MNPs magnetoplexes with and without the influence of magnetic field. In contrast, the HDR efficacy was obtained to be around 0.56% and 0.44% transfection by T3-CRISPR/Cas9-PEI-MNPs magnetoplexes with and without the influence of magnetic field, respectively (Fig. 7(ii)). Although there was no significant difference found between magnetofected and non magnetofected cells, the presence of a magnetic field might result in a slightly higher efficiency, indicating that magnetofection moved the magnetoplexes very rapidly to the cell surface. Namgung and colleagues had developed hybrid superparamagnetic iron oxide nanoparticle-branched polyethylenimine (BPEI–SPION) magnetoplexes for gene transfection of vascular endothelial cells. The BPEI–SPION showed enhanced transfection in a magnetic field after incubation of magnetoplexes for 15 min, which might be due to the rapid movement and accumulation of the magnetoplexes to the target cells^28^. Similarly, Hryhorowicz *et al*. observed improvement in transfection efficacy when utilizing PEI–Mag2 complexed with CRISPR/Cas9 plasmid nanoparticles in the presence of the magnetic field and state that the increase in transfection might be due to the acceleration of magnetic properties that aids in enhancing concentrations of magnetic transfection complexes on the cell surface^24^. Grześkowiak and colleagues presented that plasmid DNA with PEI–Mag2 nanoparticles and DF-Gold as an enhancer in combination with an applied magnetic field facilitates vector internalization, endosomal escape and protects the nucleic acid against nuclease degradation^39^. These results in conjugation with confocal microscopy provide an insight into the course of events.

### Cellular uptake of magnetoplexes and their gene expression

To further establish the rapid gene transfection and the role of magnetoplexes on cellular uptake, we designed a straightforward confocal microscopic study. The microscopic observations were in agreement with the quantitative cellular transfection efficiency data obtained by FACS (Fig. 7(i,ii)). The percentage of RFP-positive cells represents gene transfection efficiency of T0-CRISPR/Cas9-PEI-MNPs magnetoplexes (Supplementary Fig. S2(g,i)) in comparison with conventional transfection method by Lipofectamine (Supplementary Fig. S2(e)). The expression of mRFP after Lipofection of T0-CRISPR/Cas9 was observed to be around 20% whereas the T0-CRISPR/Cas9-PEI-MNPs showed transfection efficiency at around 13 and 10.3% for magnetofected and non-magnetofected mode of transfection, respectively (Fig. 7(ii)). This result verifies the penetration of the CRISPR/Cas9 magnetoplexes into the nucleus and dissociation of DNA from the magnetoplexes into the nucleus. It has been proposed that the entry of the magnetoplexes into the nucleus is assisted by the breakdown of the nuclear membrane, which occurs during the cell division^40,41^ indicating that the efficiency of gene expression also depends largely on the cells capacity to proliferate^31^. The level of genome editing efficiency at the target site for NHEJ was observed by expression of BFP, and HDR in presence of template was observed by expression of GFP (Fig. 8). The positive control T3 transfected by Lipofectamine is shown in Fig. 8a, T3-CRISPR/Cas9-PEI-MNPs magnetofected cells in Fig. 8b, and T3-CRISPR/Cas9-PEI-MNPs non-magnetofected cells in Fig. 8c, whereas Supplementary Fig. S2(d,f,h) are blank and negative controls respectively. The confocal microscopy images clearly depict the expression of respective fluorescence indicating the mode of repair mechanisms, which is in accordance with FACS analysis. Because mRFP signal depends on the number of particles per cells in contrast to gRNA-CRISPR/Cas9 where one molecule can trigger the fluorescence, we could detect some blue and green cells with almost no visible red signal.

## Conclusion

The CRISPR/Cas9 system has become widely used tool in genome editing. An efficient, non-viral delivery of CRISPR/Cas9 system to the cells would indeed improve gene therapy approaches. In this study, we showed CRISPR/Cas9-PEI-MNPs to directly deliver constructs encoding Cas9 protein and gRNA enabling site-specific incision and NHEJ or HDR correction of BFP and GFP genes respectively. The use of CRISPR/Cas9-PEI-MNPs complex in combination with an inhomogeneous magnetic field proved to be an effective alternative for rapid and non-toxic strategy to develop CRISPR/Cas9-mediated genome editing. Additional studies are needed to determine whether the utilization of MNPs adapted for delivery of a CRISPR/Cas9 genome-editing system can be used in *in vivo* conditions.

### Statistical analysis

The results are reported as means ± standard deviation. The statistical analyses between different groups were determined with a non-paired t-test. Probability (p) ≤ 0.05 was considered as significant. All statistical analyses were performed using the Originlab 8 program (OriginLab Corporation USA).

## Supplementary information

Supplementary Information.
