# Supplementary material for: Polyethylenimine based magnetic nanoparticles mediated non-viral CRISPR/Cas9 system for genome editing
Source: Sci Rep. 2020 Mar 12;10:4619. doi: 10.1038/s41598-020-61465-6 (PMC7067791; doi:10.1038/s41598-020-61465-6)
Supplement: Supplementary file 1 — Supplementary Information. [file 41598_2020_61465_MOESM1_ESM.docx]

**Supplementary:**

**Polyethylenimine based magnetic nanoparticles mediated non-viral CRISPR/Cas9 system for genome editing**

**S.S. Rohiwal^1^, N. Dvorakova^1^, J. Klima^1^, M. Vaskovicova^1^, F. Senigl^2^, M. Slouf^3^, E. Pavlova^3^, P. Stepanek^3^, D. Babuka^3^, H. Benes^3^, Z. Ellederova*^1^ and K. Stieger*^4^**

^1^The PIGMOD center, Institute of Animal Physiology and Genetics, v. v. i., The Czech Academy of Sciences, Libechov, Czech Republic, ^2^ Institute of Molecular Genetics, The Czech Academy of Sciences, Praha 4, Czech Republic, ^3^ Institute of Macromolecular Chemistry CAS, Heyrovského nám. 2, 162 06 Prague 6, Czech Republic, ^4^ Department of Ophthalmology, Justus-Liebig-University, 35392 Giessen, Germany.

*****Corresponding author:

Prof. Knut Stieger

Justus-Liebig-University Giessen, Department of Ophthalmology, Friedrichstrasse 18, 35392 Giessen, Germany, Knut.Stieger@uniklinikum-giessen.de

and

Dr. Zdenka Ellederova

The PIGMOD center, Institute of Animal Physiology and Genetics CAS, v. v. i. Rumburská 89, Liběchov 277 21, Czech Republic, ellederova@iapg.cas.cz


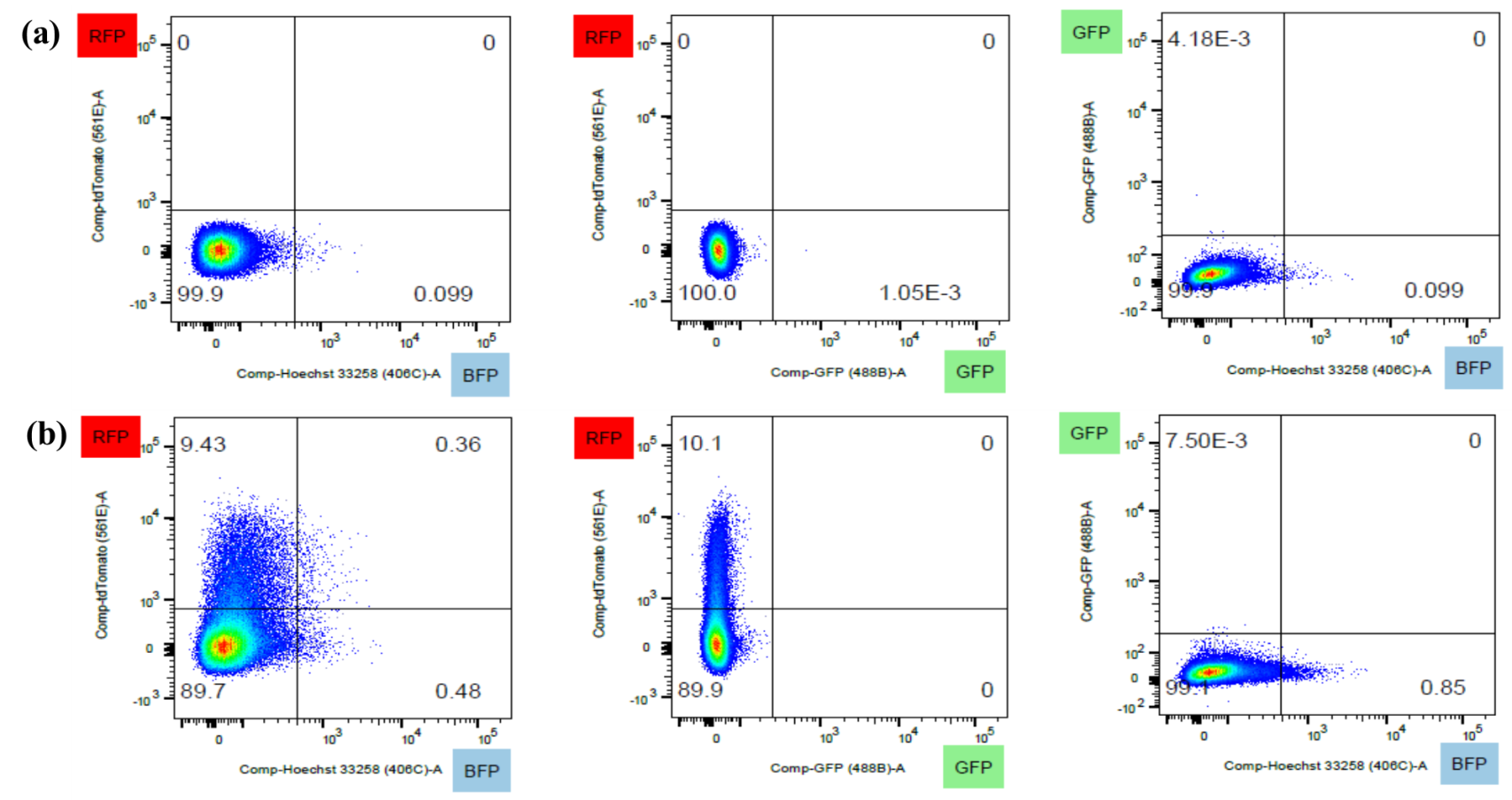
**DNA repair ability of magnetoplexes:**


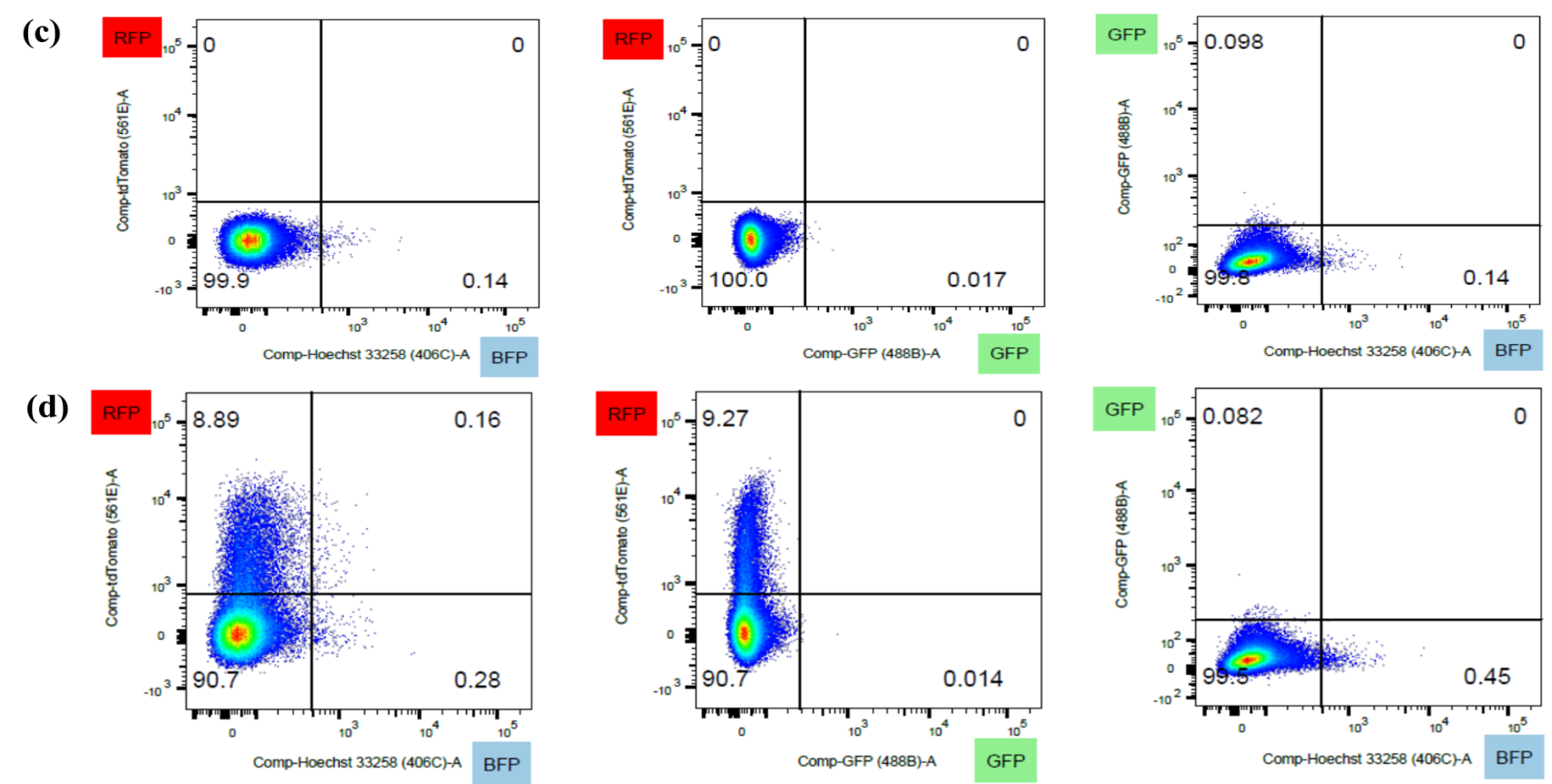


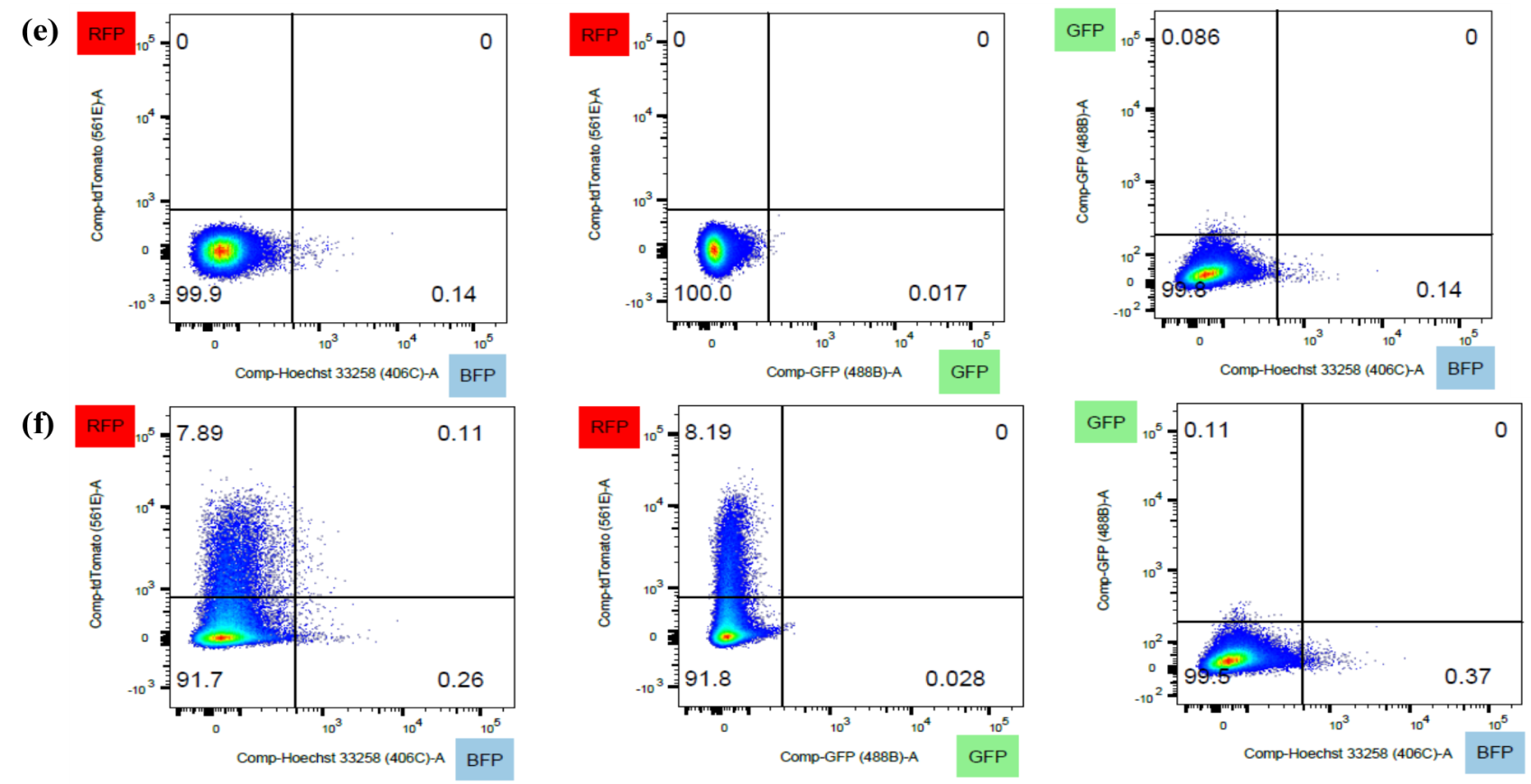


**Supplementary Figure S1.** (i) FACS representative data of the respective samples and controls in which (a) negative control (blank cells), (b) T0 positive control (Lipofectamine), (c) PEI-MNPs-M magentofected, (d) CRISPR/Cas9-PEI-MNPs-T0-M magentofected, (e) PEI-MNPs-X non-magentofected, (f) CRISPR/Cas9-PEI-MNPs-T0-X non-magentofected,

**Cellular uptake of magnetoplexes and their gene expression:**

**
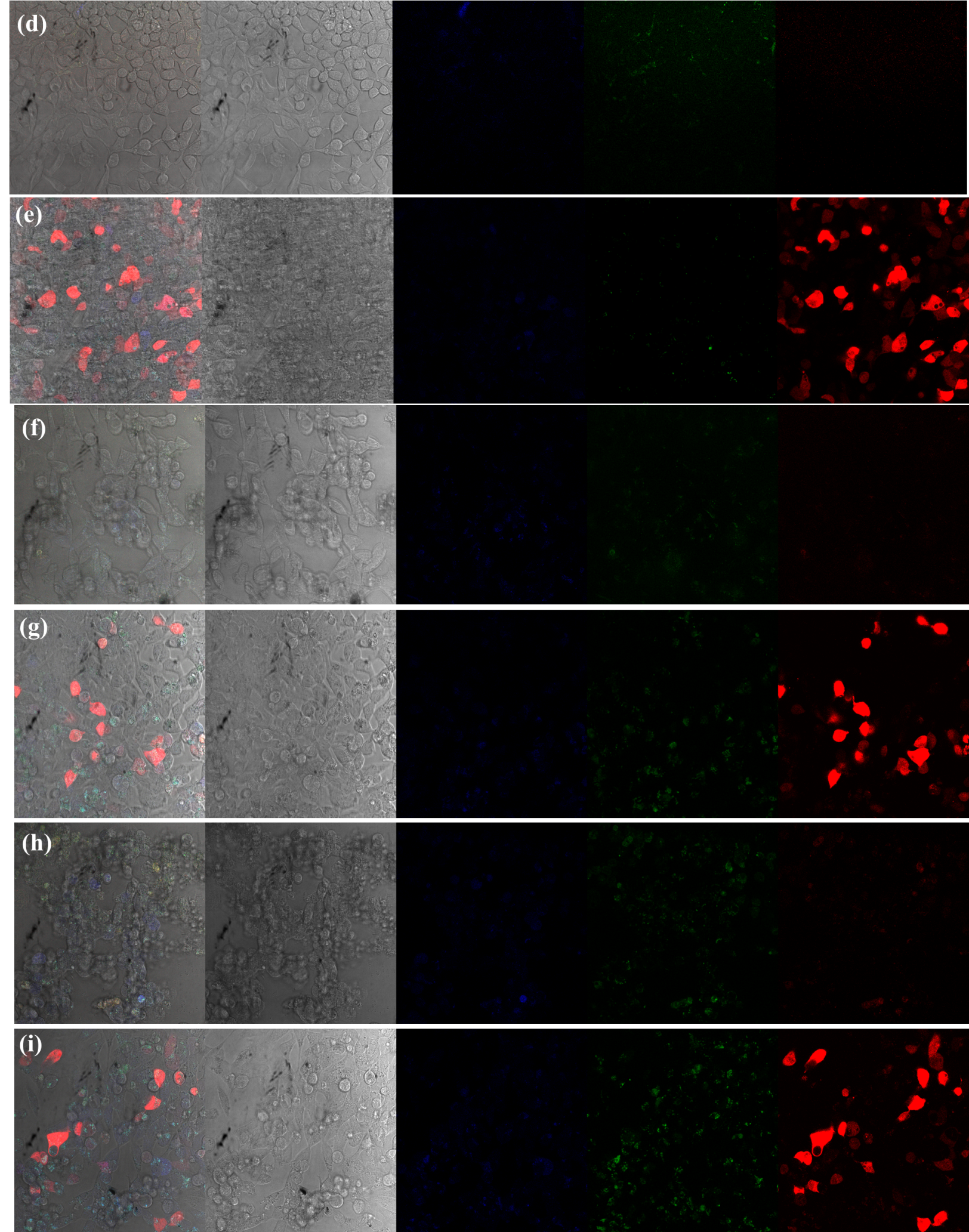
**

**Supplementary figure S2**. Fluorescent microscopy image of HEK293-TLR3 cells transfected by T0 and T3 CRISPR/Cas9-PEI-MNPs complex at N/P ratio 10 for 72 h. The percentage of transfection efficiency is determined by mRFP positive cells (a-i), the type of DNA repair mechanism NHEJ and HDR due to the presence of TLR3 sequence is visualized by BFP and GFP respectively, (d) negative control (blank cells), (e) TO positive control (Lipofectamine), (f) PEI-MNPs magnetofected, (g) CRISPR/Cas9-PEI-MNPs-TO magentofected, (h) PEI-MNPs non-magentofected and (i) CRISPR/Cas9-PEI-MNPs-TO non-magnetofected.


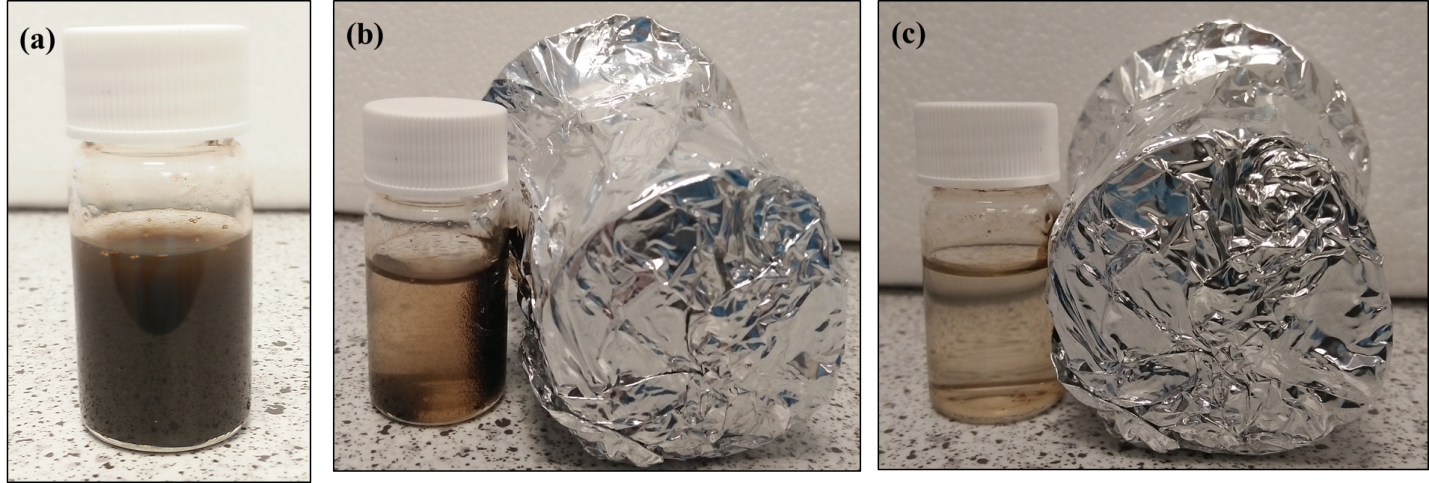
**Synthesis of Magnetic nanoparticles**

**Supplementary Figure S3**. Magnetization of superparamagnetic NPs: (a) MNPs after sonication in deionized water, (b) sequentially separating the MNPs from the aquatic environment by a magnet, (c) final magnetic separation of MNPs.


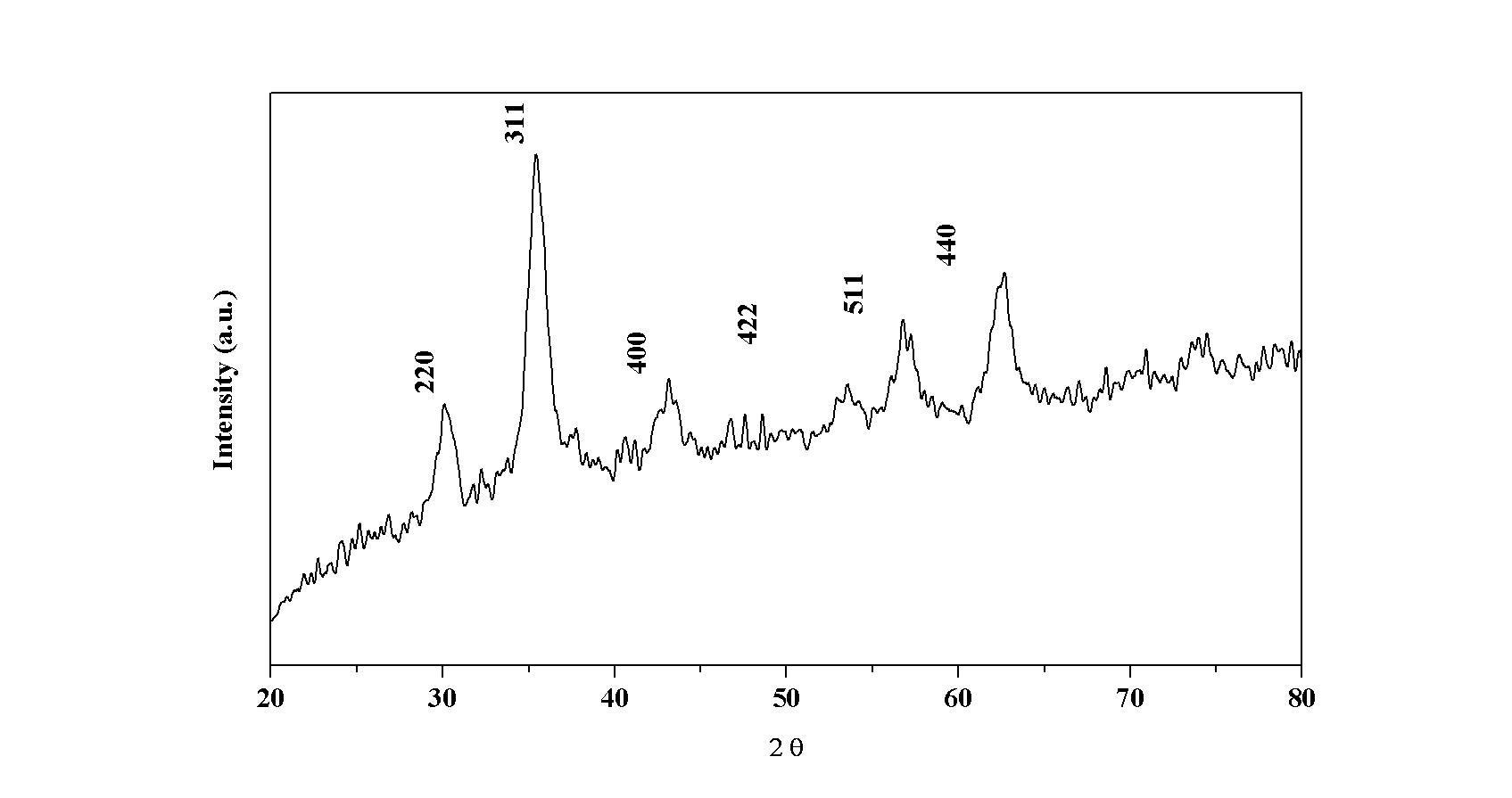
**XRD Pattern**

**Supplementary figure S4.** The XRD pattern of Fe_3_O_4_ nanoparticles

The characteristic peaks of magnetite were detected at Miller indices 220, 331, 400, 422, 511 and 440. The XRD patterns reveal highly crystalline particles with an inverse spinel structure and lattice parameters similar to those of magnetite [1]. These characteristics peak were then matched with the JCPDS file number 82-1533, which corresponds to Fe_3_O_4_ phase. The Gaussian fit of the most intense peak (311) was used to calculate the full width at half maxima for determination of crystallite size (D) by the equation D ¼ 0.9l/b cos q, where l ¼ 2.2897 A, the wavelength ˚ of incident X-ray, q is the corresponding Bragg's diffraction angle and b is full width at half maxima of the (311) peak. The average crystallite size of bare Fe3O4 MNPs found to be 12 nm.

**References:**

[1] P. Guardiaa, B. Batlle-Brugala, A. Rocab, O. Iglesiasa, M. Moralesb, C. Sernab, A. Labarta and X. Batllea, J. Magn. Magn. Mater., 2007, 2, 316
